# Benzodiazepines I: Upping the Care on Downers: The Evidence of Risks, Benefits and Alternatives

**DOI:** 10.3390/jcm7020017

**Published:** 2018-01-30

**Authors:** Jeffrey Guina, Brian Merrill

**Affiliations:** 1 Department of Psychiatry, University of Michigan Medical School, Ann Arbor, MI 48109, USA; 2 Department of Psychiatry, Wright State University Boonshoft School of Medicine, Dayton, OH 45435, USA; brian.merrill@wright.edu

**Keywords:** benzodiazepine, sedative, hypnotic, anxiolytic, psychopharmacology, alprazolam, clonazepam, evidence-based

## Abstract

Benzodiazepines are some of the most commonly prescribed medications in the world. These sedative-hypnotics can provide rapid relief for symptoms like anxiety and insomnia, but are also linked to a variety of adverse effects (whether used long-term, short-term, or as needed). Many patients take benzodiazepines long-term without ever receiving evidence-based first-line treatments (e.g., psychotherapy, relaxation techniques, sleep hygiene education, serotonergic agents). This review discusses the risks and benefits of, and alternatives to benzodiazepines. We discuss evidence-based indications and contraindications, and the theoretical biopsychosocial bases for effectiveness, ineffectiveness and harm. Potential adverse effects and drug-drug interactions are summarized. Finally, both fast-acting/acute and delayed-action/chronic alternative treatments for anxiety and/or insomnia are discussed. Response to treatment—whether benzodiazepines, other pharmacological agents, or psychotherapy—should be determined based on functional recovery and not merely sedation.

## 1. Introduction

Benzodiazepines (BZDs) are some of the most commonly prescribed medications in the world [1,2]. However, they are commonly used in ways not supported by the literature. Here, we review their historical and common uses, and the evidence for and against the use of benzodiazepines.

### 1.1. History of Benzodiazepines

The history of sedative-hypnotics extends back thousands of years to the early use of alcohol. Likely because of its history and cultural perceptions, alcohol has not been categorized as a sedative-hypnotic in the *Diagnostic and Statistical Manual of Mental Disorders (DSM)* [3]. However, many consider alcohol to be a sedative-hypnotic because of its mechanism of action, synergy and cross-tolerance with barbiturates and BZDs, and very similar intoxication and withdrawal phenomena.

Since the 1800s, several other sedative-hypnotics have been developed. After the introduction of chlordiazepoxide in 1960, BZDs quickly became the most common “minor tranquilizer,” replacing older agents like alcohol, chloral hydrate, paraldehyde, barbiturates and meprobamate. Compared to these agents, BZDs were considered to have less side effects, toxicity, abuse (i.e., continued use despite substance-related problems), physical dependence (i.e., tolerance and/or withdrawal), and suicide potential. Unlike barbiturates, which were commonly abused (often intravenously to provide a “rush”) and are lethal at levels 10 times the usual therapeutic dose, BZDs have a therapeutic ratio above 100 [4]. While physicians recognized that BZDs did “not represent a panacea for all neurotic ills … does not eliminate the causes of intra- or extrapsychic stress … do not directly affect the psychodynamic and environmental factors responsible for emotional problems … do not, at least in a direct sense, affect the characterologic or personality side of the patient,” many were excited about the hope that they could “facilitate problem solving, contribute to the de- and reconditioning of emotional responses, and enable the patient to cope with intra- and extrapsychic stress more appropriately” in conjunction with psychotherapy [5]. Many prescribers were led to believe that BZDs were harmless and had no dependence risk [1,4]. BZDs were prescribed frequently and often long-term for various conditions: Anxiety, insomnia, substance withdrawal, anesthesia, muscle tension, seizures, psychosis, depression, narcosynthesis (aka the “BZD interview”) for combat neurosis, ordinary life stressors, vague psychosomatic complaints, and even arrhythmias and myocardial infarction [1,5,6,7,8,9]. By the 1970s, BZDs were the most commonly prescribed medications in the world [1].

Unfortunately, the potential for abuse and dependence was rapidly discovered [10]. In 1975, BZDs were placed on the Food and Drug Administration (FDA) restricted drug list, reflecting growing concerns about abuse [5]. After years of patients and clinicians anecdotally reporting tolerance and withdrawal with long-term use of even therapeutic doses, several controlled trials in the 1980s confirmed that BZDs can cause dependence [1]. In 1990, the American Psychiatric Association (APA) officially recognized the risk of BZD dependence [4,11]. With growing data and warnings about BZDs as well as the arrival of safer, more effective anti-anxiety medications like selective serotonin reuptake inhibitors (SSRIs) and the popularization of cognitive behavior therapy (CBT), BZD use slowly declined after the mid-1980s (mostly due to the reduction of BZD use as anxiolytics as there was little change in BZD use as hypnotics) [12,13]. In recent years, the Department of Veterans Affairs (VA) has been particularly driven to replace BZD prescriptions with evidence-based treatments, resulting in a significant reduction in BZD prescriptions from 1999 to 2009 [14]. VA Researchers [14] speculated that “ideally, benzodiazepine use has decreased because veterans with Posttraumatic stress disorder (PTSD) are receiving higher rates of evidence-based treatments for core symptoms, including cognitive-behavioral therapy and adequate trials of first-line medications, thereby minimizing residual symptoms and eliminating the need for adjunctive benzodiazepines.”

Despite recommendations against long-term BZD use (i.e., more than 2–4 weeks), many providers continue to prescribe them for months or even years, allowing for dependence and diversion to occur [1,15]. Total BZD use actually increased from 1999 to 2014, largely driven by increases in long-term use [16]. For example, as many as 94% of PTSD patients using BZDs receive at least 30 day supplies and about two-thirds receive at least 90 day supplies [14,17]. About 15% of the U.S. population take BZDs in any given year and about 6% of the US population have abused sedative-hypnotics [12], drugs that—unlike cannabis, amphetamines and opioids—exclusively originate from the medical system.

### 1.2. Common Uses Today

Since the introduction of SSRIs, BZDs are mostly used for anesthesia, alcohol or BZD withdrawal, and the symptomatic treatment of anxiety and/or insomnia [18,19,20]. Many BZDs received FDA approval (see Table 1) for the treatment of “anxiety states” or “anxiety disorders” prior to disorders having specific diagnostic criteria first present in *DSM-III* in 1980. Therefore, BZD treatment represents an off-label use (i.e., without FDA disease-specific approval) for most mental disorders. This is especially true for PTSD, which was first officially recognized in *DSM-III* and was separated from anxiety disorders in *DSM-5* in 2013. Though the only FDA approved medications for PTSD are sertraline and paroxetine (both antidepressants), of PTSD patients receiving pharmacotherapy, 65–90% receive antidepressants, 37–74% receive sedative-hypnotics and 21–34% receive antipsychotics [20,21,22]. Therefore, PTSD patients were frequently prescribed medications not supported by evidence-based clinical practice guidelines (CPGs), which generally discourage BZDs and marginally support atypical antipsychotics. Most studies about BZD prescriptions are in participants with PTSD (likely because of the VA’s increased interest in evidence-based treatment for veterans, who commonly have trauma-related and substance-related disorders), but one can imagine that there are parallels in prescribing across diagnoses.

There are four typical groups of BZD users: Older medically ill patients who are taking many medications including BZDs usually prescribed by non-psychiatrists, panic disorder or agoraphobia patients, patients with recurrent dysphoria with vague and chronic symptoms, and patients with chronic sleep disturbances. The latter two groups have been found to be much more likely than the first two groups to misuse BZDs (e.g., take with other substances like alcohol) [11]. Factors increasing the likelihood of an individual being prescribed BZDs include: Comorbid psychiatric disorders (especially anxiety disorders, PTSD, and borderline personality disorder) [17,20,22,23,24,25], substance use disorders (SUDs) [20,22,23,26], inpatient psychiatric treatment [20,24], longer duration of disorder [14,17], more VA disability compensation [17,20], concurrent opioid prescriptions [23,26], concurrent prescriptions for other BZDs [26], and number of traumas [25]. Meanwhile, decreased BZD prescription for PTSD patients correlates with increased prescription of antidepressants [24,27], trazodone [17], prazosin, quetiapine, and non-BZD hypnotics [27]. While correlations with psychotherapy have not been studied, one study predicted that the “expanded use of high-quality, evidence-based psychotherapy for PTSD, insomnia, and other comorbidities could have contributed to declining reliance on benzodiazepine prescribing” [23]. Studies conflict about howgender [17,22,23,26] and age [17,20,22,23,26] affect BZD prescriptions.

When first-line serotonergic agents are initiated for anxiety, it is not uncommon for prescribers to co-administer BZDs, despite this practice not being supported by the evidence and posingrisks [2,19,28]. Serotonergic agents (e.g., SSRIs, serotonin and norepinephrine reuptake inhibitors [SNRIs]) are the first-line pharmacologic treatments for anxiety disorders, including panic disorder (PD) and generalized anxiety disorder (GAD) [29]. These “antidepressants” (that are not only effective for depression) typically take four to six weeks before they exert clinical effect, though in the treatment of anxiety symptoms this delay can be even longer [30]. Further compounding this delay is that antidepressants are not necessarily effective at starting doses and therefore during titration to an effective dose a patient can remain symptomatic. Consequently, it can be months before anxiety relief is experienced from an antidepressant regimen. BZDs are commonly used as adjuncts during the first few weeks of starting a serotonergic agent with the hopes that once a therapeutic dose is achieved, the BZD can be discontinued [31,32,33]. Unfortunately, there is no evidence to support this practice, the combination may increase adverse effects without increasing therapeutic effects, and it is not uncommon for patients to continue BZDs long-term in the presence or absence of the antidepressant [2,34]. BZDs do not make SSRIs more effective when prescribed simultaneously, despite conventional wisdom. There are also no long-term benefits, but there is a long-term risk of physical dependence (i.e., tolerance and/or withdrawal), associated with prescribing BZDs when initiating SSRIs [2,12]. Despite many clinicians intending to taper/discontinue BZDs after the 4–6 weeks it takes SSRIs to have their therapeutic effect, 12% of patients receiving this regiment continue BZDs for over 6 months—sometimes in the absence of SSRIs—likely indicating the difficulty of discontinuing BZDs once started. Despite the absence of efficacy and the evidence of risks, the rate of physicians prescribing this way almost doubled between 2001 and 2014 [2].

It is common for psychiatrists to lament referrals from primary care after the primary care prescriber initiated BZDs and then expects for the psychiatrist to assume responsibility for the medication. Contrary to the prediction of the authors of one large study (*n* = 356,958) that primary care providers were responsible for most BZD prescriptions, mental health providers prescribed BZDs more than twice as often as other providers [21]. However, the study also found that BZD prescription rates were highly variable between facilities. This conforms with suggestions that facility culture can influence prescribing practices, and that a lack of awareness of CPGs and evidence is one of the most common barriers to best practices. Provider and facility characteristics are an important factor in BZD prescribing. Professional development activities, lectures, journal club presentations and informal interactions among physician peers may influence local culture at the facility level and encourage evidence-based practices [17]. However, the study [21] acknowledged the difficulty prescribers face when they “inherit” patients who previously received BZDs from other prescribers (many of whom started BZDs prior to CPGs), creating “an immediate tension” as providers attempt to balance individual patient factors with the need to provide evidence-based care.

## 2. Evidence for Benzodiazepine Prescription

### 2.1. Evidence-Based Indications

The only mental disorders—not including alcohol/sedative-hypnotic withdrawal and catatonia—for which there is an evidence basis for BZDs are PD, GAD, social anxiety disorder (SAD), and insomnia [8,31,32,33,34,35,36,37]. For these four conditions, BZDs have only demonstrated efficacy and are only recommended for short-term durations (i.e., less than 2–4 weeks) and for treatment-resistant cases (i.e., after failing multiple first-, if not second-, line treatments).

Sedative-hypnotics can provide rapid relief for symptoms like anxiety and insomnia. Because many anxiety and insomnia patients will be seen in primary care and medical specialty settings, it is important that all providers be familiar with first-line evidence-based treatment recommendations. BZDs are intended to be used for no more than 2–4 weeks in the treatment of *severe* anxiety and/or insomnia that are resistant to multiple evidence-based treatments. Some argue to “limit benzodiazepine prescriptions to emergency situations where a rapid symptomatic amelioration is required before the onset of action of antidepressant treatment allows the reduction and finally the cessation of benzodiazepines” [35].

Systematic reviews and CPGs generally recommend psychotherapy (e.g., CBT) and serotonergic agents (e.g., SSRIs, SNRIs) as first-line treatments for anxiety disorders [19,33,38,39,40,41,42,43,44]. However, because not all individuals respond or fully remit, or have adverse effects with these treatments, it is important to know options for those who have continuing symptoms or do not tolerate first-line treatments [45]. BZDs are an evidence-based option for PD, GAD, and SAD patients who fail psychotherapy and multiple serotonergic agents, or require adjunctive treatment when serotonergic agents are partially effective but other adjuncts have failed.

In general, it is helpful, prior the onset of treatment, to determine if the impairment caused by an anxiety disorder would be best ameliorated with short- or long-term treatment. For instances of anxiety that are caused by infrequent, predictable inciting stimuli (e.g., fear of air travel, public speaking or dental procedures) short-term BZD treatment may be the simplest, safest, and most effective course of action. Though certainly non-BZD options should be considered (e.g., propranolol for performance anxiety) as these options may have less potential for toxicity (e.g., cognitive dulling which would make it difficult to get to or think through a performance) and physical dependence. Where the experience of anxiety is more enduring (e.g., GAD, PD) long-term pharmacologic treatment may be useful.

### 2.2. Theoretical Basis

Sedative/anxiolytic (i.e., anxiety-reducing) and hypnotic/soporific (i.e., sleep-inducing) effects occur because BZDs potentiate the effects of gamma-aminobutyric acid (GABA), the primary inhibitory neurotransmitter in the central nervous system (CNS). BZDs exert their effects allosterically on GABA-A receptors by binding the BZD receptor complex. All BZDs interact with this complex and are capable of producing both hypnotic and anxiolytic effects, despite specific FDA indications for particular disorders. Therefore, selection of a particular agent for a given patient largely depends upon pharmacokinetic, rather than pharmacodynamic properties [46,47]. When BZDs interact with GABA-A receptors, ion channels open more frequently, increasing the inflow of chloride ions which increases membrane polarization and inhibits neuron firing [4]. The result is CNS depression [12].

Some have postulated that underlying differences in GABA receptors can predispose individuals to develop anxiety disorders, and/or that trauma/stressors can induce modulation or downregulation of GABA receptors in those that develop anxiety disorders [48]. Dysfunction in the GABA system (including BZD-GABA binding) in different brain areas have been theorized to correlate with common anxiety symptoms: Amygdala (emotional modulation dysfunction and fear conditioning), prefrontal cortex (attention and working memory deficits), hippocampus (memory formation dysfunction), and striatum (salience reactivity and misinterpretation of aversivestimuli) [48,49,50]. While GABA receptors are distributed throughout the CNS, the primary desired target for BZD activity for anxiolytic effects is the amygdala and related circuits (e.g., the locus ceruleus which is implicated in autonomic cardiovascular activity, periaqueductal gray which is implicated in autonomic motor activity, the parabrachial nucleus which is implicated in autonomic respiratory activity, and hypothalamus which is implicated in the endocrinological stress response and sleep regulation) [51]. Therefore, it is theorized that BZDs can normalize these brain areas in individuals with hypoactive GABA activity (e.g., provide inhibition in a hyperactive amygdala), thus relieving anxiety and improving sleep.

## 3. Evidence against Benzodiazepine Prescription

While there is some evidence for the efficacy of short-term BZDs in treatment-resistant cases of PD, GAD, SAD and insomnia, BZDs are frequently overprescribed for other indications for which there is no evidence of efficacy, to individuals who have contraindicated comorbid conditions, for longer periods of time than are recommended, and before other first and second-line treatments are tried [52,53,54,55,56,57].

### 3.1. Conditions and Durations with No Evidence of Efficacy

Even in the conditions for which BZDs have proven efficacy—PD, GAD, SAD and insomnia—there is no evidence of long-term efficacy.

Aside from PD, GAD, SAD and insomnia, no other mental disorders have an evidence-basis for BZDs. To the contrary, PTSD [31,36,44,58] and phobias [59] have evidence of ineffectiveness or even harm. Biological explanations for BZD-induced anxiety include discontinuation symptoms (i.e., withdrawal or rebound symptoms resulting from GABA receptor desensitization and glutamate receptor sensitization) and/or worsening of underlying anxiety pathophysiology (e.g., disrupting normal hypothalamic-pituitary-adrenal [HPA] axis stress responses, and inhibiting serotonin regulation) [58]. BZDs have demonstrated the ability to interfere with fear extinction [17,23,39], which is critical for the improvement of anxiety and is likely why BZDs have been found to increase fear conditioning with phobias [59] and to have fear-sensitizing effects in response tostress [23,60,61]. For many, BZDs—especially when used long-term—actually worsen anxiety.

BZD treatment for PTSD is particularly concerning because BZDs have no known preventative value for PTSD and may actually increase the risk of developing PTSD 2–5 times among those with trauma; BZD side effects overlap with several PTSD core symptoms (e.g., avoidance, negative mood, inattention, amnesia, recklessness, irritability); PTSD is commonly comorbid with conditions that are contraindicated for BZDs (e.g., SUD, traumatic brain injury [TBI], depression); and BZDs can inhibit trauma-focused psychotherapy by promoting avoidance of exposure, numbing trauma-related emotions, and inhibiting of cognitive processing (all of which is necessary for recovery) [3,58,62,63,64,65]. For these reasons and the lack of any study supporting BZD efficacy for PTSD symptoms, most CPGs (e.g., American Psychiatric Association, British Association for Psychopharmacology, International Consensus Group of Depression and Anxiety, International Society for Traumatic Stress Studies, Veterans Affairs/Department of Defense) recommend against the use of BZDs for PTSD [43,44,66,67,68]. Berger et al. [45] summarized: “There is no compelling scientific evidence of the effectiveness of benzodiazepines either in the prevention of PTSD or in the treatment of its core symptoms although clinical experience suggests that they may improve sleep and agitation, at least in the short term. These limited advantages must be weighed against the marked potential for addiction that characterizes this class of drugs, particularly considering that PTSD patients have higher rates of drug abuse/dependence than the general population”.

There are no studies outside of case reports that support the use of BZDs as adjunctive or symptomatic treatments. Additionally, some worry that “because benzodiazepines reduce anxiety without addressing the underlying PTSD, clinicians may incorrectly believe the patient has improved, thus delaying definitive PTSD care” [39]. High rates of comorbidity suggest that PTSD and SUDs are functionally related, a concept supported by several studies indicating a pathway (likely related to corticotropin-releasing hormone and norepinephrine) whereby PTSD precedes substance use [69]. Substance use is commonly a direct result of PTSD and often correlates with the amount of terror felt during trauma [7]. In one large study (*n* = 274,297), while the strongest diagnostic predictor of sedative-hypnotic prescription for PTSD was a comorbid anxiety disorder, 77.2% of those receiving sedative-hypnotics did not have a comorbid anxiety disorder [20] and, therefore, did not have an indicated diagnosis. Many researchers, clinicians and organizations have recommended that pharmacotherapy—BZDs or otherwise—should not be used as a routine first-line PTSD treatment [33].

There are no studies to support the long-term use of BZDs for insomnia, and the evidence that is supportive of short-term use is based on very few studies (which were insufficient to perform a meta-analysis of triazolam) but triazolam was not considered first-line due to a higher risk for “rebound anxiety” [37,57]. Some insomnia studies using BZDs demonstrate short-term efficacy without evaluating long-term efficacy, some demonstrate no benefit after one day to several months of use, and several demonstrate no statistical difference between BZDs and placebo even in the short-term [13,37,58,70,71,72]. Likely explanations for the lack of long-term efficacy of BZDs for insomnia include rebound insomnia, tolerance, and sleep architecture dysregulation. BZDs can directly worsen sleep by disrupting sleep architecture, which may continue even months after discontinuation [4]. Several studies have demonstrated that even short-term BZD use can decrease sleep time, decrease deep-stage/slow-wave sleep, increase rapid eye movement (REM) sleep latency, increase stage 2 non-REM sleep and decrease delta count [1,12,72,73]. Like alcohol, the sedating properties of BZDs often deceive patients into thinking that BZDs improve sleep when, in actuality, they tend to induce the early stages of sleep while inhibiting the deep, most restorative, stages of sleep. Fortunately, BZDs do not permanently alter sleep architecture, as after an initial decrease in sleep time during acute withdrawal, sleep parameters significantly improve following BZD discontinuation to levels not significantly different from healthy controls [1,12,72].

### 3.2. Contraindicated Comorbid Conditions

While receiving BZDs for conditions without an indicated diagnosis is concerning, receiving BZDs when contraindications are present is dangerous. Mental conditions for which BZDs are contraindicated include SUDs, depression, suicidal ideation, bipolar disorder, psychosis, and neurocognitive disorders (NCDs) including those due to TBI [58,74]. Unfortunately, each of these have high comorbidity with anxiety disorders, for example: SUD (33–50%) [58,75], depressive disorders (10–55%) [3], and TBI (4–48%) [3,76]. Even more concerning, several studies have demonstrated high rates of sedative-hypnotics among patients with contraindicated comorbid psychiatric disorders: Depressive, substance use [20,22] and neurocognitive disorders [20,22,77]. In fact, anxiety patients with these contraindicated disorders are usually more likely to receive sedative-hypnotics [20,22,23,26,77].

Because the development of BZD use disorder (BUD) with prescribed medications occurs in at least 50% of patients with preexisting or active SUDs [1], BZDs are almost universally considered contraindicated for patients with any history of SUDs (except in cases of acute alcohol or sedative-hypnotic withdrawal).

Comorbid depression is particularly problematic. Because of high rates of comorbidity and the large overlap of symptoms (e.g., negative thoughts, insomnia, irritability, fatigue, suicidality) with depressive and anxiety disorders [19,35,38,66,78], a missed diagnosis of depression is possible when treating only anxiety or insomnia. When the diagnosis is missed, a prescriber may be less cautious than necessary about providing a BZD. BZDs do not prevent the development of depression, can be depressogenic and can lead to suicide attempts [8,42,70,79,80]. BZDs have the potential to cause or worsen depression, dysphoria, dissatisfaction with life, and suicidal thoughts and behaviors [39,58,62,81]. Risk factors for attempted and completed suicide while using BZDs include BZD intoxication, repeated BZD intoxication, high doses of prescribed BZDs, and underlying depression [3,23,82]. While the anxiolytic and hypnotic effects of BZDs diminish as tolerance develops, depression and impulsivity with high suicidal risk commonly persist and are “often interpreted as an exacerbation or as a natural evolution of previous disorders and the chronic use of sedatives is overlooked” [8]. Unlike antidepressants, in which activating effects usually improve with continued treatment, BZD-induced depression is unlikely to improve. This is part of the reason BZDs may be indicated for anxiety disorders like GAD and PD, but not for PTSD, which is not a pure anxiety disorder as it also has features of depressive disorders and has a different underlying pathophysiology. Potential explanations for BZD-induced depression include general CNS depression, prefrontal cortex [PFC] inhibition (hypoactive PFC is implicated in depression, disinhibition and suicidality), BZD-induced inhibition of central monoamine activity including serotonergic and noradrenergic, and withdrawal dysphoria [58,83,84,85]. While BZD-induced depression may be exacerbated after discontinuation, evidence suggests that mood significantly improves after the acute withdrawal period to levels better than during use [8]. Modern practice guidelines (e.g., APA, The Department of Veterans Affairs and the Department of Defense guidelines (VA/DoD)) for the treatment of major depressive disorder either suggest using buspirone as an augmentation agent for anxiolysis or evaluating the treatment response of an antidepressant prior to BZD trials [86]. Sedating antidepressants (e.g., doxepin, mirtazapine, trazodone, and amitriptyline) can be effective for anxiety and/or insomnia especially with comorbid depression [57]. Fortunately, the first-line treatments are the same for anxiety disorders, PTSD, and depressive disorders: Psychotherapy and serotonergic agents.

In addition to the mental conditions above, BZDs have several other contraindications: BZD hypersensitivity, pulmonary disease (e.g., sleep apnea, chronic obstructive pulmonary disease), Parkinson’s disease, porphyria, hepatic disease, renal impairment, closed-angle glaucoma, myasthenia gravis, children and adolescents less than 18 years old, pregnancy, and breast-feeding [74]. Sleep apnea is of particular concern because it can be comorbid with or mimic anxiety disorders (e.g., insomnia, fatigue, anxiety, and irritability). Similarly, pulmonary diseases in general, Parkinson’s disease and porphyria are each associated with anxiety. Finally, obesity is a concern due to being associated with reduced elimination of BZDs [74].

### 3.3. Adverse Effects

BZDs are linked to a variety of adverse effects, whether used long-term, short-term, or as needed. While BZDs would be ideal anxiolytics if they could selectively inhibit the hyperactive amygdala (implicated in anxiety), because GABA receptors are widely distributed throughout the CNS, BZDs indiscriminately target the entire brain. This is particularly problematic for those areas of the brain that are already hypoactive in anxiety disorders, such as the PFC (implicated in mood dysregulation including depression and anxiety, behavioral dysregulation including disinhibition and irritability, and cognitive dysfunction including inattention and cognitive processing of risk/stress/trauma) and hippocampus (implicated in amnestic effects and inhibited fear extinction). However, global CNS inhibition also leads to adverse effects via every area of the brain including motor, sensory, speech, and respiratory impairments [19,58,74,85]. Table 2 attempts to summarize an exhaustive list of cognitive, emotional, behavioral, perceptual and physical adverse effects from BZDs [1,3,4,8,11,15,24,31,59,74,78,79,80,83,87,88,89,90,91].

#### 3.3.1. Vulnerable Populations

Adverse effects are more likely to occur with elderly patients, NCDs, TBI, hepatic impairments, renal impairments, pulmonary impairments (e.g., sleep apnea) and in combination with other CNS depressants (e.g., alcohol, sedative-hypnotics, opioids, antihistamines, anticonvulsants, neuroleptics and sedating antidepressants) [15,83].

Extra caution is recommended when prescribing BZDs to the elderly. Several considerations are relevant to this recommendation. Specifically, disinhibition, falls, and respiratory depression are of concern in the elderly given the higher prevalence of other chronic medical conditions. The elderly are particularly sensitive to BZDs because of pharmacokinetic changes that prolong medication effects (i.e., distribution, metabolism, elimination), increased use of additional medications that can compound adverse effects, and increased presence of even minimal brain damage [8,13,73]. People of advanced age may have impaired hepatic metabolism and this could lead to the accumulation of benzodiazepines and their metabolites in the plasma and consequent toxicity. The lengthy half-life of diazepam often makes that agent more risky in the elderly. Many clinicians favor the so-called “L.O.T. drugs” in this population (lorazepam, oxazepam, and temazepam) as they do not require oxidative metabolism in the liver and have no active metabolites. Agitation, associated with dementia is a common reason for medical intervention in the elderly and benzodiazepines may be useful, especially in situations where anxiety is prominent. Short-term use is the recommendation with special care needed to use only the lowest effective dose. BZD toxicity in the elderly can cause cognitive impairment even with short-term therapeutic doses [11]. After nicotine and alcohol, BZDs are associated with the greatest risk of SUD in the elderly [13].

Additional concerns for women of child-bearing age include teratogenicity and breast-feeding. BZDs have been labeled class D teratogens and are contraindicated in breast-feeding mothers [83]. The risk of BZDs in pregnancy is not entirely clear. There is evidence that BZDs cross the placenta to the fetus and are secreted in breast milk to infants, although studies are mixed on specific consequences. Early evidence suggested a heightened risk of facial anomalies though this finding has not been consistently found and may have been confounded by other predisposing teratogenic etiologies (e.g., anti-epileptic drugs) [92]. BZD use during pregnancy may cause preterm birth, low birth weight, neonatal withdrawal, respiratory depression, temperature dysregulation, apnea, hypotonia, poor feeding, sedation, lethargy, weight loss, cleft palates, cardiac malformations, neural tube defects, limb deficiencies, anal atresia, floppy baby syndrome, lower Apgar scores, and impede the progress of childbirth [15,31,93,94,95]. BZD use during breastfeeding may cause sedation, weight loss, respiratory depression (especially with long-acting BZDs), and withdrawal (especially with short-acting BZDs) in children [93]. Thus, as with other clinical scenarios, the use of BZDs should include an assessment of the risks and benefits, and in pregnancy the consideration of risks should be extended to the unborn child. This includes the risks and benefits of prescribing BZDs and the risks of untreated mental illness in pregnancy, which can carry a host of downstream effects. Complicated mental health in pregnancy represent an appropriate time to engage a psychiatrist for collaboration or consultation.

#### 3.3.2. Tolerance

A major disadvantage with BZDs is that tolerance to their therapeutic effects develop relatively quickly while many adverse effects persist. Tolerance develops to hypnotic effects within days to weeks, to myorelaxant effects within weeks, to anticonvulsant effects within weeks to months, and to anxiolytic effects within months [1,8,83,96]. This explains why patients commonly increase dosage over time and many eventually take more than one type of BZD after the first loses effectiveness [1,3,4,73,79]. While therapeutic effects are subject to tolerance, adverse effects often are not, with cognitive, depressogenic, and disinhibiting effects typically persisting long-term when present and increasing in risk if doses are increased in response to therapeutic tolerance [8]. Because tolerance to brain stem depressant effects develop more slowly than tolerance to sedating effects, patients may take more BZDs to achieve desired effects (e.g., hypnotic, anxiolytic or even euphoria in recreational abusers) which may cause sudden respiratory depression, hypotension and/or death [3].

BZD tolerance develops as the result of long-term alterations of the intraneural gene expression and function of GABA receptors, likely including downregulation of BZD binding sites on GABA-A receptors (similar to alcohol), uncoupling of the allosteric linkage of the BZD-GABA receptor complex, and changes in receptor subunit turnover [1,4,8,83,96]. Chronic BZD-induced enhancement of GABA also likely results in a compensatory sensitization of excitatory systems (including glutamate) [1,97].

#### 3.3.3. Cognitive, Emotional and Behavioral Adverse Effects

Many adverse effects of BZDs overlap with symptoms associated with mental disorders (see Table 3 and Table 4). This overlap may make it difficult to determine if symptoms are due to underlying anxiety disorders or BZDs. Symptoms with specificity for sedative-hypnotic toxicity include nystagmus, slurred speech and unsteady gait [4]. Overlapping symptoms and adverse effects indicate that BZDs may synergistically worsen underlying anxiety (which may be misattributed to a primary worsening of underlying anxiety). Giving credence to this concern, several studies have demonstrated that psychiatric symptoms, cognitive performance and general physical health improve after BZD discontinuation (especially in the elderly) [1].

Mental health patients commonly have inattention (e.g., GAD, PTSD, depression), and amnesia (e.g., PTSD, dissociative disorders). Therefore, it is particularly disturbing that cognitive impairments are among the adverse effects caused by BZDs (i.e., benzodiazepine-induced neurocognitive disorder). While cognitive effects can occur as part of BZD intoxication, withdrawal or delirium, they can also occur as a direct effect of BZDs [3]. Cognitive impairments are more common with high doses and/or long-term use of BZDs, but can also be caused by low doses, short-term use and even single daily doses (especially in the elderly) [3,13]. While the most common cognitive adverse effects of BZDs (sedation and drowsiness) often improve as tolerance develops, many cognitive impairments persist with continued use: Attention, concentration, learning, working memory, episodic memory, semantic memory, verbal memory, nonverbal memory, procedural memory, general intelligence, problem solving, verbal reasoning, procedural reasoning system, speed of processing, sensory processing, visuospatial abilities, motor performance and psychomotor speed [1,6,8,12,15,71,83]. In the elderly, these symptoms may be mistaken for an unrelated progressive dementia [1,3]. In fact, BZDs can cause dementia which persists after discontinuation of use [3,98]. In fact, elderly patients often attribute memory problems to age rather than BZDs and about 10% of elderly patient referred to memory clinics have cognitive impairments that are substance-induced, often due to BZDs [13]. Risk factors for BZD-induced neurocognitive disorder include older age (especially persistent use after 50 years old) [3,6,12,23], other substance use (especially alcohol), other NCDs (especially TBI), delirium (in which even very low doses of BZDs may be intoxicating), longer use and primary psychiatric disorders (especially PTSD, psychotic, depressive and bipolar disorders) [3]. For these and other reasons, BZDs are contraindicated in patients with a history of SUD, NCD, TBI and depression. Finally, some have argued that BZDs need to be discontinued prior to psychotherapy because the cognitive effects of BZDs decreased psychotherapy effectiveness, likely due to reduced ability to cognitively process and remember material presented in therapy [45]. Fortunately, several studies have demonstrated that cognition improves after discontinuing BZDs (especially working and episodic memory, visuospatial search abilities, information processing, attention and simple reaction time) [13]. While cognition usually slowly recovers after BZD discontinuation, like with alcohol, these effects can persist after an extended period of abstinence (i.e., benzodiazepine-induced persisting neurocognitive disorder, previously called benzodiazepine-induced persisting amnestic disorder) [1,3,6,12].

BZD use for anxiety disorders can be problematic because anxiety, panic attacks, phobias and social avoidance are among the adverse effects of BZDs. While BZDs may provide short-term relief for anxiety, there is often a paradoxical long-term worsening of anxiety. Similar concepts have been studied with nicotine (which is associated with worse anxiety with chronic use and improvement after discontinuation) [99,100] and opioids (i.e., opioid-induced hyperalgesia, in which chronic opioid use makes patients more sensitive to pain) [101,102]. Nevertheless, patients often believe these substances are helping, mistaking the temporary relief of withdrawal symptoms for improvement of baseline symptoms. It seems patients are much more adept at detecting the immediate impact of a BZD on anxiety symptoms as opposed to detecting the long-term deterioration in mood or worsening of anxiety. Potential explanations for BZD-induced anxiety disorder include: Rebound or withdrawal anxiety (i.e., after tolerance develops to the anxiolytic effects of BZDs, they no longer treat baseline anxiety but only treat discontinuation symptoms at best returning anxiety levels back to their pre-BZD baseline) [1,8]; BZDs increasing fear responses after stressful exposures likely by fear-sensitization and generalizing conditioneddefeat [60,61,103,104]; BZDs being associated with a loss of self-confidence in one’s ability to cope with stressors without medications [83]; and BZDs indirectly worsening anxiety by promoting avoidance, preventing fear extinction, inhibiting cognitive processing of past traumas and present risk assessment, and inhibiting the process of desensitizing individuals to anxiety through both therapeutic and natural exposure [7,17,23,25,27,39,58,59]. During the 1980s, many behavioral therapists and researchers began realizing that BZDs reduce the therapeutic effects of exposure. This undercut the purpose of exposure therapy: Gradually decrease anxiety through repeated exposure to anxiety-provoking stimuli. It also challenged an assumption made since the 1960s that a drug-induced low-anxiety state accompanying anxiety-provoking stimuli would lead to faster fear extinction. Several studies suggest that “patient attribution of fear reduction to the drug rather than to improvement in coping may result in a loss of gains after withdrawal of the drug” [59]. Therefore, BZDs may directly worsen avoidance and indirectly worsen anxiety by inhibiting emotional and memory processing during exposure (therapeutic or natural), decreasing learning efficiency and decreasing fear extinction. Avoidance is an important consideration because, although patients often present complaining about arousal and autonomic symptoms, it is arguably the most disabling, the most difficult to treat, and the most predictive of poor outcome. Once physical dependence develops, the affective states that are numbed with sedatives become more intense during withdrawal states causing patients to wrongly believe that the withdrawal state is their baseline and believe that they need the substance. While anxiety may be exacerbated during acute withdrawal, evidence suggests that patients have significantly less anxiety five weeks after discontinuation then they had during BZD use (when users often subjectively report being less anxious than they were prior to using), and avoidance and social functioning improve within one year of BZD discontinuation [1,8].

Insomnia is one of the most common chief complaints of patients with mental disorders, and sleep deprivation can cause fatigue, irritability, inattention, depression, anxiety, dysfunction, and reduced quality of life [21,63,73]. Therefore, it is particularly disturbing that BZD-induced sleep disorder, insomnia and nightmares are among the adverse effects of BZDs. While BZDs may provide short-term relief for insomnia, there is often a paradoxical long-term worsening of sleep. After tolerance develops to the hypnotic effects of BZDs, it is likely that they no longer treat baseline insomnia but only treat rebound and withdrawal insomnia (i.e., benzodiazepine-induced sleep disorder), at best allowing the patient to sleep as they did prior to BZD use [1,8,11]. Many patients claim they cannot sleep without BZDs, not realizing they are only preventing rebound insomnia caused by chronic BZD use. One randomized control trial concluded “ineffectiveness of BZDs in aiding sleep is also suggested by the significantly positive correlations between measures of BZD intake (dose, cumulative dose) and patient’s ratings of sleeping problems. In the withdrawers at baseline, the higher the nightly dose of BZD the greater the rating of sleep problems” [13]. In addition to rebound insomnia, BZDs often exacerbate sleep disturbances by directly disrupting sleep architecture (like alcohol). Sleep architecture may continue to be disrupted even months after BZD use [4]. Several studies have demonstrated that even short-term BZD use can worsen several sleep parameters: Decreasing sleep time, increasing rapid eye movement (REM) sleep latency, decreasing slow wave sleep (stages 3 and 4 non-REM sleep), increasing stage 2 non-REM sleep and decreasing delta count [1,12,72,73]. After an initial decrease in sleep time during acute BZD withdrawal, sleep parameters significantly improve (especially in the elderly) to levels not significantly different from healthy controls: Sleep quality, increased slow wave sleep and increased delta count [1,8,12,13,72]. This indicates that BZD use can cause poor sleep, but sleep usually recovers after discontinuation.

Many patients with mental health problems struggle with irritability, aggression, violence, suicidality, and/or self-destructive or reckless behavior. Therefore, it is particularly disturbing that the list of potential BZD adverse effects include all of these, as well as poor judgment and behavioral disinhibition. While BZDs may provide acute improvement of agitation, there is often the potential for worsening with regular use. While alprazolam has the strongest link with aggression, it may occur with any BZD (including the supposedly “safe” clonazepam) [105]. A large long-term cohort study concluding that “our results provide additional support for discouraging long-term benzodiazepine use to manage core symptoms of PTSD” [106]. In addition to rebound irritability, BZDs can cause “paradoxical reactions” (e.g., disinhibition, impulsivity, excitement, irritability, aggression, hostility, rage attacks, violence, homicidal ideations, suicidality) [24,83,105]. The proposed mechanisms of BZD-induced violence is similar to that of alcohol, including PFC inhibition and systemic inhibition of serotonin (which is associated with anger, impulsivity and violence at low levels) [84]. While evidence suggests that BZD-induced violence is rare, it is usually severe when it does occur [105]. Like with alcohol, BZD-induced disinhibition can result in subsequent interpersonal (e.g., arguments, fights, and inappropriate sexual or aggressive behavior), medical (e.g., physical risks like fights, and driving an automobile or operating a machine while impaired) and/or legal problems (from the above as well as neglect of children) [3]. Risk factors for paradoxical reactions with BZDs include other substance use (especially alcohol) [24,90,105], neurocognitive disorders (especially TBI) [17,84,105], a history of disinhibition or impulsivity (especially with borderline personality disorder) [39,105,106], anxiety disorders [24,90], older age [31], learning disabilities [105], and motivational drive [107]. Unfortunately, some of these factors (substance use, anxiety and personality disorders) also correlate with increased BZD prescription for PTSD patients. For these and other reasons, BZDs are contraindicated in patients with a history of SUD, TBI or a history of impulsivity or violence [39,44]. However, there are reports of patients engaging in uncharacteristic behaviors (e.g., aggressive, destructive, sexually inappropriate and criminal) that only occur after BZD use, without any history of similar behaviors, and usually improve after discontinuation [15,90,105]. While the anxiolytic and hypnotic effects of BZDS disappear as tolerance develops, irritability, anger and impulsivity with high suicidal risk commonly persist [8]. Furthermore, BZD-induced irritability may be exacerbated during withdrawal, evidence suggests that irritability significantly improves after the acute withdrawal period to levels better than during use [8,13].

#### 3.3.4. Physical Adverse Effects

Sedation (including daytime drowsiness from a previous night’s dose) is the most common BZD adverse effect [12]. Other common adverse effects include unsteadiness, impaired psychomotor speed and accuracy, cognitive impairment and slurred speech. BZDs can be fatal, whether directly by overdose (usually due to synergistic respiratory depression when combined with other CNS depressants) or indirectly by injury (usually as the result of BZD-induced cognitive impairment, inattention, incoordination, unsteadiness, delayed reaction time, visuospatial impairments, behavioral disinhibition and/or poor judgment) [4]. BZDs—from both nonmedical use and use as prescribed—are associated with emergency department visits, typically due to overdoses (whether suicidal, accidental and/or recreational) or injuries (e.g., falls, fights, motor vehiclecollisions) [1,3,11,13,23,31,71,105]. BZDs increase the risk of fractures 50–110%, with no difference in risk between short-acting and long-acting BZDs, and combining BZDs with other substances more than doubles the risk of injury [12]. These risks are particularly disturbing for anxiety patients, for which comorbid substance use is common and who often already have difficulty concentrating, irritability or impulsivity.

#### 3.3.5. Dependence, Misuse, Abuse and Addiction

BZDs have been shown to be relatively efficacious for the short-term treatment of GAD, PD, SAD and insomnia, but long-term efficacy is unproven while the risk of physical dependence and addiction is. “Like alcohol, these agents are brain depressants and can produce similar substance/medication-induced and substance use disorders” [3]. There has been much debate and confusion about what constitutes a BZD use disorder in patients prescribed BZDs. Some have tried to clarify this by distinguishing non-mutually exclusive terms: Physical dependence, misuse, recreational abuse and addiction. The *DSM-5* nomenclature is consistent with these distinctions for prescribed medications (e.g., opioid analgesics, sedatives, stimulants), exempting tolerance and withdrawal from the diagnostic criteria for SUD when these are the only symptoms present and medications are taken as prescribed [3]. Below we define and differentiate various substance-related terms.

Physical dependence is the presence of tolerance and/or withdrawal. Of those prescribed therapeutic doses of BZDs long-term, 58–100% inadvertently become physically dependent (therapeutic dose dependence) [1,12]. Iatrogenic dependence is more likely to occur with high doses and/or short acting BZDs, but can occur with any BZD prescription [1,26,31]. As tolerance develops, some patients are able to persuade providers to increase their dose (prescribed high-dose dependence). Even patients who recognize the long-term ineffectiveness of BZDs often continue to take them to stave off withdrawal symptoms, which are often mistaken for baseline anxiety [1]. With increased usage, tolerance and the potential for withdrawal worsen. Both BZD tolerance and withdrawal can be explained by the chronic desensitization of GABA receptors and sensitization of glutamate receptors. Considering that low GABA receptor sensitivity and hyperactive glutamate are implicated in anxiety [1,97], this would imply that chronic BZD use could actually exacerbate the pathophysiology of anxiety rather than improves it.

Prescription medication misuse is the use of medications differently than prescribed (e.g., higher doses, increased frequency, combined with alcohol, etc.), obtaining medications from several providers without informing them of the others (i.e., “doctor shopping”), or diversion (i.e., giving, selling or trading medications to others). Misuse is more likely to occur with the elderly, chronic pain and a history of SUD [91]. BZDs can cause drug reinforcement and misuse in patients without histories of SUD in conditions of continuous BZD availability, especially in patients with anxiety, insomnia and histories of moderate alcohol consumption [91]. As BZD tolerance develops, there is often a gradual increase in the frequency of self-administration beyond provider recommendations, which may be subtle and mistaken for appropriate use [91]. Some patients combine BZDs with alcohol when they are not able to obtain desired therapeutic effects due to tolerance [83]. “The individual is likely to continue to justify use on the basis of his or her original symptoms of anxiety or insomnia, but substance seeking behavior becomes more prominent, and the individual may seek out multiple physicians to obtain sufficient supplies of the medication” [3]. While some solely rely on providers for BZDs, others obtain extra medications from the black market (generally supplied by patients who divert prescriptions) [1].

Recreational abuse is the intentional use of medications to produce intoxication (a “high”). Short-acting BZDs are more likely to be used for intoxicating purposes, although long-acting BZDs (including the supposedly “safe” clonazepam) can also produce a high [3,83]. BZDs can be used as the primary drug of choice, but they are used in conjunction with other substances about 80% of the time [1,83]. In combination with other CNS depressants, like alcohol, cannabis or opioids (including methadone), BZDs are often used by substance users to enhance intoxication (i.e., a “boost”) [3,4,83]. These combinations can be lethal, whether by accidental or deliberate overdose [3]. Concurrent alcohol use is particularly concerning because of availability, cross-tolerance with BZDs, and because providers are commonly unaware of alcohol use [12]. This is a problem because patients prescribed BZDs for alcohol-induced anxiety or sleep disorders (whether the provider recognizes that the symptoms are alcohol-related or not) are at increased risk of misusing BZDs [3,4]. Many substance users also use BZDs to alleviate unpleasant effects associated with other substances, including self-treating opioid withdrawal (i.e., a “fix”) and stimulant intoxication (i.e., a “come down”) [1,3,83]. The combination of stimulant-induced wakefulness/agitation and BZD-induced cognitive impairments (e.g., judgment, memory) can result in unpredictable behavior [4]. Recreational abusers are more likely to use BZDs by insufflation (“snorting”) or intravenously (which is associated with its own inherent medical risks, like infections) [1,3,15,105]. Higher doses, combining BZDs with other substances, and non-oral use all increase the risk of overdose.

Addiction is a chronic pattern of compulsive drug-seeking associated with a loss of control, preoccupation and anticipation, and continued use despite psychosocial dysfunction anddistress [3,8,12,108]. Table 4 describes BUD and related disorders. When in the context of only taking BZDs as prescribed, BUD should only be diagnosed when these factors are present (i.e., not based on tolerance or withdrawal alone) [3]. Risk factors for developing BUD include pre-existing or active SUDs (especially alcohol, sedative-hypnotics, cannabis, opioids and stimulants), family history, medical availability, early onset of use, chronic medical conditions, chronic insomnia, chronic dysphoria, impulsivity, and borderline or dependent personality disorders [1,3,8,11,12,17,90]. Unfortunately, some of these factors—particularly SUDs and borderline personality disorder—also correlate with increased BZD prescriptions [20,22,23,25,26]. BUD is almost universally iatrogenic due to widespread over-prescription [1]. Because BZDs are exclusively manufactured by pharmaceutical companies (unlike most other substances), the prevalence of dependence correlates with its medical availability [3,4]. In addition to prescriptions, BZDs can be obtained through the internet, the black market (“the street”) or theft (e.g., from manufacturers, delivery services, hospitals, clinics, pharmacies or patients’ medicine cabinets).

### 3.4. Drug–Drug Interactions

Pharmacodynamically, the most significant concern with BZDs is CNS/respiratory depression. Therefore BZDs are contraindicated with other CNS depressants. This is particularly concerning since many people with anxiety have comorbid SUD, often with CNS depressants like alcohol, opioids, ketamine, and marijuana. The CNS depressants considered to have “major” interactions (i.e., concomitant use may cause respiratory depression, hypotension, profound sedation and death) are: Barbiturates (such as butalbital, which is sometimes found in headache medicine), cannabinoids, ethanol, melatonin (which has also been found to increase BZD binding at receptor sites), opioids and opiate agonists (e.g., tramadol, buprenorphine), pramipexole, and sodium oxybate [74]. Despite these contraindications, significant predictors of BZD prescription rates include concurrent opioid analgesic [23,26] and concurrent BZD prescriptions [26]. “Moderate” interactions include: Anticonvulsants, antihistamines and antipsychotics (all of which are commonly used in mental health patients). Regarding opioids and BZDs, one should typically be discontinued during the administration of the other. A common scenario is a patient undergoing both alcohol/sedative withdrawal and opioid withdrawal, during which the former is life-threatening and the latter is not, and therefore a BZD taper should be favored over buprenorphine/methadone administration. However, the risks of delaying opioid agonist treatment often outweighs the concerns about synergistic CNS depressant effects. When the risks of delay outweigh the benefits, opioid dependent patients may be relatively safely stabilized with agonists while also tapering benzodiazepines. Because of the exponential risk of respiratory depression and death with concomitant use of BZDs and opioids these agents should only be used together with abundant caution (e.g., prescribed at lower than usual doses) and only after exhausting other options. Medications such as pseudoephedrine, phenylephrine and caffeine may have minor-moderate drug interactions with BZDs because they antagonize the sedative effects of BZDs [74], which could lead to some patients taking more BZDs to counteract the stimulants.

BZDs can be differentiated based on pharmacokinetic properties. Lorazepam, oxazepam and temazepam are only metabolized via glucuronidation but not oxidation through the Cytochrome P450 (CYP) pathway. Otherwise, BZDs are CYP3A4 substrates, though some can also be CYP2C19 substrates and CYP3A4 inhibitors. Medications that have a “major” potential to increase BZD levels are: Aprepitant, atazanavir, boceprevir, ceritinib, chloramphenicol, cobicistat, conivaptan, darunavir, dehydroepiandrosterone, delavirdine, fluvoxamine, fosamprenavir, fosaprepitant, grapefruit juice, idelalisib, imatinib, indinavir, itraconazole, ketoconazole, lopinavir, nefazodone, nelfinavir, ritonavir, saquinavir, telithromycin, and tipranavir [74]. Of this list, fluvoaxime and nefazodone are particularly concerning as they are psychotropic medications. Conversely, BZDs may increase the levels of: Cisapride (arrhythmia risk) and lomitapide (hepatotoxicity risk). Finally, BZD levels may be reduced by: St. John’s Wort (an over-the-counter psychotropic commonly used for anxiety and/or depression).

## 4. Alternatives

BZDs are often used as first-line treatments for anxiety and insomnia despite evidence supporting equal or superior efficacy of other treatment modalities that are less associated with toxicity (e.g., behavioral therapy for insomnia, antidepressants for anxiety) [52,53,54]. There are several evidence-based alternatives for BZDs for both fast-acting/acute and chronic uses. Regardless, of the treatment used, response should be monitored and determined based, not only on self-reported symptom changes but also functional improvement. Recovery should be equated with “adaptation, reappraisal, and learning” [7]. Reduced anxiety and increased sleep in the presence of sedative-induced emotional numbness and detachment, and sedative-facilitated avoidance of healthy relationships and employment do not denote recovery.

### 4.1. First-Line Treatments for Anxiety and Insomnia

Psychotherapy (e.g., CBT, exposure, relaxation, eye movement desensitization and reprocessing (EMDR)) is the gold standard treatment for anxiety while medications are generally considered adjunctive (only serotonergic agents are considered first-line pharmacologic monotherapies) [36,44,55]. Nevertheless, patients with anxiety are frequently prescribed medications that are not evidence-based or supported by CPGs, and BZDs are often initiated before evidence-based treatments are even tried or offered. While serotonergic agents have stronger efficacy for anxiety than BZDs, it is notable that a 2016 Cochrane review [56] concluded insufficient evidence exists to compare antidepressants to BZDs for the treatment of PD in terms of efficacy and tolerability.

For insomnia, psychological and behavioral therapies (e.g., CBT including CBT for insomnia (CBT-I), stimulus control, relaxation, sleep restriction) are the standard of care. Medications, at best, received a “consensus” or “weak” recommendation—each the lowest recommendation from respective insomnia CPGs [37,57]—with temazepam and triazolam being the only BZDs to receive such a recommendation and, then, only for short-term use. Nevertheless, insomnia patients are frequently prescribed BZDs before gold standard treatments (i.e., psychological and behavioral) or even other evidence-based medications (e.g., ramelteon, doxepin, suvorexant) [37,57].

Because of the wide spectrum of symptoms and the varying pathophysiological components behind mental disorders, there are no panaceas. However, while first-line treatment (e.g., psychotherapy, SSRIs) fail to control all symptoms in all people, unlike BZDs, they do not exacerbate the symptoms and disorders they are not effective in treating.

### 4.2. Additional Pharmacologic Alternatives

Serotonergic agents (e.g., SSRIs, SNRIs, tricyclics, mirtazapine, monoamine oxidase inhibitors, trazodone, nefazodone, buspirone) have the strongest evidence of greater therapeutic effects and less adverse effects than BZDs, but adrenergic inhibitors (e.g., propranolol, prazosin, clonidine, guanfacine), antihistamines (e.g., hydroxyzine, diphenhydramine), anticonvulsants (e.g., gabapentin, pregabalin, lamotrigine, topiramate, valproate), antipsychotics (e.g., quetiapine, olanzapine, risperidone), memantine and triiodothyronine all have stronger evidence for certain anxiety/insomnia disorders than BZDs [19,31,33,35,36,38,39,40,41,42,43,44,45]. Consideration for unique adverse effects and comorbidities should be given. For example: Hydroxyzine may be particularly effective for people with anxiety, insomnia, nausea/vomiting and/or allergies (all indications); amitriptyline may be particularly effective for people with anxiety, insomnia, depression, neuralgias and/or migraines (all indications); clonidine may be particularly effective for people with anxiety, insomnia, inattention +/− attention-deficit hyperactive disorder, tics, cancer-related pain, and/or hypertension (all indications); lamotrigine may not be first-line but may be used earlier in the course of successive medication trials for people with anxiety, bipolar disorder (especially bipolar depression), and epilepsy (both indications), and/or obesity (lack of weight gain effects); and it may be prudent to avoid (or try later in the course of medication trials) olanzapine in those with obesity and/or diabetes (metabolic risks) but useful to try it earlier in those with anxiety, insomnia, depression, psychosis and/or bipolar disorder.

## 5. Conclusions

Overwhelming evidence for or against the use of BZDs is lacking for most psychiatric disorders. What is known is the potential for rapid anxiolysis and sedation with these medications, though long-term, outcome-based studies are needed. The potential for abuse and addiction is high with BZDs and should be a consideration each time they are prescribed. Due to the lack of evidence of efficacy and presence of evidence of many risks, BZD prescription is only recommended in severe, disabling anxiety or insomnia. Until questions about long-term BZD use are satisfactorily addressed, “the wise prescriber will limit his prescriptions in number to patients who are severely anxious or insomniac; in dosage to the lowest effective; and in duration to a few weeks rather than months or years” [15]. Long-term BZD use has no efficacy and significant harm. “The risk/benefit ratio of these drugs becomes less favorable or even adverse as treatment becomes prolonged: Efficacy wanes and risks accumulate” [15]. Risks include dependence, withdrawal, “chronic subtle toxicity and the interference with the underlying psychopathology” [8]. Their greatest asset is also their greatest liability: Drugs that work immediately tend to be addictive” [82]. Without strong evidence of efficacy and with significant evidence of risks, a variety of evidence-based treatments should be considered prior to initiating BZD trials. Assessment of recovery should be based on improved functioning and not merely self-reported sedation (which often does not correlated with functional recovery).

## Figures and Tables

**Table 1:** Food and Drug Administration (FDA) approvals for benzodiazepines.

Benzodiazepine	Anxiety	Insomnia, Short-Term	Alcohol Withdrawal	Seizure	Other
flurazepam		+			
chlordiazepoxide	+		+		Preoperative anxiety
diazepam	+		+	+	Muscle spasms, preoperative sedation
clorazepate	+		+	+	
clobazam					Lennox-Gastaut syndrome (adjunct)
clonazepam	+			+	Panic disorder; periodic limb movement disorder; neuralgia
estazolam		+			
temazepam		+			
lorazepam	+	+		+	Preoperative sedation; chemotherapy-related nausea/vomiting
oxazepam	+		+		
alprazolam	+				Panic disorder
triazolam		+			
midazolam					Procedural sedation

**Table 2:** Benzodiazepine adverse effects that overlap with core symptoms, associated symptoms and common comorbidities of PTSD.

Benzodiazepine Adverse Effects	Overlap with Anxiety	Example of Mental Disorders with Similar Symptoms
Physical		
dizziness (vertigo, lightheadedness/syncope)	+	panic, somatic, dissociative
nausea/vomiting	+	panic, somatic
speech (dysarthria, slurred)		
motor (incoordination/unsteadiness, ataxia, poor reaction time, tremors, weakness, hyperreflexia, nystagmus, diplopia)	+	panic, somatic
restlessness/psychomotor agitation	+	GAD, PTSD, mood, somatic
muscle pain/tension/spasms	+	GAD, PTSD, somatic
sensory (visuospatial impairments, photophobia, hyperacusis, strange tastes, tinnitus, paresthesia/ numbness/burning)	+	panic, somatic
coryza/nasal congestion		
rash	+	somatic
sexual (dysfunction, menstrual irregularities)	+	somatic, depressive, SUD, sexual
blood dyscrasias		
autonomic (brady- vs. tachy-cardia, hypo- vs. hyper-tension, dyspnea/ respiratory depression vs. hyperventilation, diaphoresis, fever/hyperpyrexia)	+	panic, PTSD, somatic
dependence (tolerance, withdrawal)		SUD
seizures	+	Somatic
Cognitive		
sedation/drowsiness/fatigue	+	GAD, depressive, SUD, NCD
inattention/poor concentration	+	GAD, depressive, SUD, NCD
nightmares/intrusive thoughts	+	PTSD
memory impairments/amnesia	+	PTSD, dissociative, SUD, NCD
impaired judgment		SUD, NCD, psychotic, bipolar
perceptual (illusions, dissociation, de-realization, hallucinations)	+	PTSD, panic, psychotic, dissociative, SUD, NCD
suicidal ideations		depressive, SUD, personality
homicidal ideations		SUD, personality
paranoia/hypervigilance	+	PTSD, SUD, psychotic
delirium/stupor/coma		SUD, NCD
Emotional		
depression/dysphoria	+	PTSD, depressive, SUD
numbness/emotional anesthesia	+	PTSD, depressive, SUD, dissociative
anger/irritability/mood lability	+	PTSD, GAD, SUD, personality, bipolar
anxiety/phobias/panic	+	anxiety, PTSD, SUD
excitement/activation/euphoria	+	anxiety, SUD, bipolar
mania		SUD, bipolar
Behavioral		
appetite/weight (anorexia, weight gain)	+	depressive, eating, SUD
insomnia	+	PTSD, GAD, depressive, SUD
avoidance/agoraphobia	+	anxiety, PTSD
impulsivity/disinhibition	+	PTSD, SUD, personality, bipolar
suicidality	+	PTSD, depressive, SUD, personality
aggression/hostility/rage/violence	+	PTSD, SUD, personality, bipolar
abuse/misuse/drug reinforcement	+	PTSD, SUD, personality

**Table 3:** Benzodiazepine-induced disorders in the Diagnostic and Statistical Manual of Mental Disorders, 5th Edition [3].

	Disorder with Onset During Intoxication	Disorder with Onset During Withdrawal	Persisting Disorder
Psychotic Disorder	+	+	
Bipolar Disorder	+	+	
Depressive Disorder	+	+	
Anxiety Disorder		+	
Sleep Disorder	+	+	
Sexual Dysfunction	+	+	
Delirium	+	+	
Neurocognitive Disorder	+	+	+

**Table 4:** Benzodiazepine-related disorders in the Diagnostic and Statistical Manual of Mental Disorders, 5th Edition.

Benzodiazepine Use Disorder	Benzodiazepine Intoxication	Benzodiazepine Withdrawal
Duration of at least 2 signs/symptoms within a 12-month period	Onset during or shortly after use	Onset following cessation or reduction in prolonged use
(1) use larger amounts or over a longer period of time than intended (2) desire to cut down use (3) a great deal of time spent obtaining (4) cravings (5) failure to fulfill major role obligations (6) use despite social or interpersonal problems (7) important activities are given up or reduced (8) use in physically hazardous situations (9) use despite physical or psychological problems (10) tolerance (11) withdrawal	“clinically significant maladaptive behavioral or psychological changes (e.g., inappropriate sexual or aggressive behavior, mood lability, impaired judgment)” at least 1 sign/symptom: (1) slurred speech (2) incoordination (3) unsteady gate (4) nystagmus (5) impaired cognition (e.g., attention, memory) (6) stupor or coma	“clinically significant distress or impairment in social, occupational, or other important areas of functioning” at least 1 sign/symptom: (1) autonomic hyperactivity (e.g., sweating, tachycardia) (2) hand tremor (3) insomnia (4) nausea or vomiting (5) transient visual, tactile, or auditory hallucinations or illusions (6) psychomotor agitation (7) anxiety (8) grand mal seizures
